# Hailey‐Hailey Disease: An Updated Review With a Focus on Therapeutic Mechanisms

**DOI:** 10.1002/hsr2.71061

**Published:** 2025-07-14

**Authors:** Mahesh Mathur, Sumit Paudel, Nabita Bhattarai, Sambidha Karki, Sandhya Regmi

**Affiliations:** ^1^ Department of Dermatology College of Medical Sciences Teaching Hospital Bharatpur Nepal

**Keywords:** ATP2C1 mutation, familial benign chronic pemphigus, general dermatology, genodermatosis, Hailey‐Hailey disease

## Abstract

**Background:**

Hailey‐Hailey disease (HHD), or familial benign chronic pemphigus, is a rare autosomal dominant genodermatosis characterized by chronic, painful, erythematous, erosive plaques and fissures at sites of friction such as the neck, axilla, groin, and perineum. The pathogenesis is due to a mutation in the ATP2C1 gene, which encodes the human secretory‐pathway calcium/manganese‐ATPase isoform 1 (hSPCA1) that regulates calcium and manganese concentration in the Golgi apparatus. The diagnosis relies on clinical presentation and characteristic histopathological features, notably a “dilapidated brick wall” appearance. There is no cure for this genodermatosis and main aim of management in HHD is to control symptoms and reduce recurrence. Multitude of topical, systemic agents, procedural therapy such as laser therapy and surgery for the treatment of HHD have been reported in the literature.

**Aims:**

This comprehensive review aims to discuss the efficacy of current HHD treatments with special focus on therapeutic mechanisms.

**Conclusion:**

This review highlights clinical and histological features of HHD and offers guidance for dermatologists involved in managing this distinct dermatosis.

## 1 Introduction

Hailey‐Hailey disease (HHD), or benign familial pemphigus, is a rare autosomal dominant genodermatosis initially described by the Hailey brothers in 1939 [1]. The disease is characterized by vesicopustules, erosions, fissures, and erythematous plaques in intertriginous areas of the body that commonly presents in the third to fourth decade of life [2]. HHD is caused by a mutation in the ATP2C1gene that alters the homeostasis of calcium ions, resulting in abnormal keratinocyte adhesion and suprabasilar acantholysis [2]. The dermatosis follows a chronic course with episodes of relapse and remission that greatly impact the patient's quality of life [3]. Despite advancements in our knowledge about the molecular basis of HHD, management remains challenging because of the lack of specific treatment protocols and controlled clinical trials [3]. This review provides a narrative review on epidemiology, pathophysiology, clinical presentation, differential diagnosis, histology, and management of HHD with a special focus on emerging treatment options.

## 2 Epidemiology

The prevalence of HHD is around 1:50,000, with no gender or race predilection [4]. The age of onset is usually in the third to fourth decade of life, but the disease may rarely present in infants or old age [5, 6].

## 3 Pathogenesis

HHD is caused by a heterozygous mutation in the ATP2C1 gene, located on the long arm of chromosome 3 (3q21‐q24). This gene encodes the human secretory‐pathway calcium/manganese‐ATPase isoform 1 (hSPCA1), an ATPase responsible for transporting Ca^2+^ and Mn^2+^ in the Golgi apparatus, thereby promoting calcium influx into this organelle and reducing its cytoplasmic level [7]. 250 missense, nonsense, frameshift, and splice‐site mutations of this gene have been described in the literature [8]. Nonsense mutations are believed to result in the reduction or absence of hSPCA1 synthesis due to mRNA degradation (haploinsufficiency). Missense mutations lead to changes in the structure, location, and stability of the hSPCA1 protein, reducing its expression and functionality. Additional factors such as heat, friction, or infection are likely to trigger the dermatosis as presentation is late and localised. Mutations of the ATP2C1 gene lead to cytosolic accumulation of Ca^2+^ which alters junctional protein synthesis, leading to acantholysis. The alteration also results in reduced mitochondrial ATP, disorganization of the actin fibers, increased oxidative stress, and reactive oxygen species, all of which impact the proliferation and differentiation of keratinocytes [3, 8].

HHD is an autosomal dominant blistering disorder with complete penetrance and variable expressivity [3]. The mutation is sporadic in 15%‒30% of cases, meaning that the affected patient has no family history of the disease [8]. In addition to the Mendelian inheritance pattern, postzygotic mutations can alter one of the alleles in a normal embryo, causing segmental lesions, that is, type 1 mosaicism, or lead to the loss of the normal allele in an embryo with a germline mutation in heterozygosity, resulting in the early segmental manifestation, later associated with the classic HHD, that is, type 2 mosaicism [9].

## 4 Clinical Features

**Figure 1:**
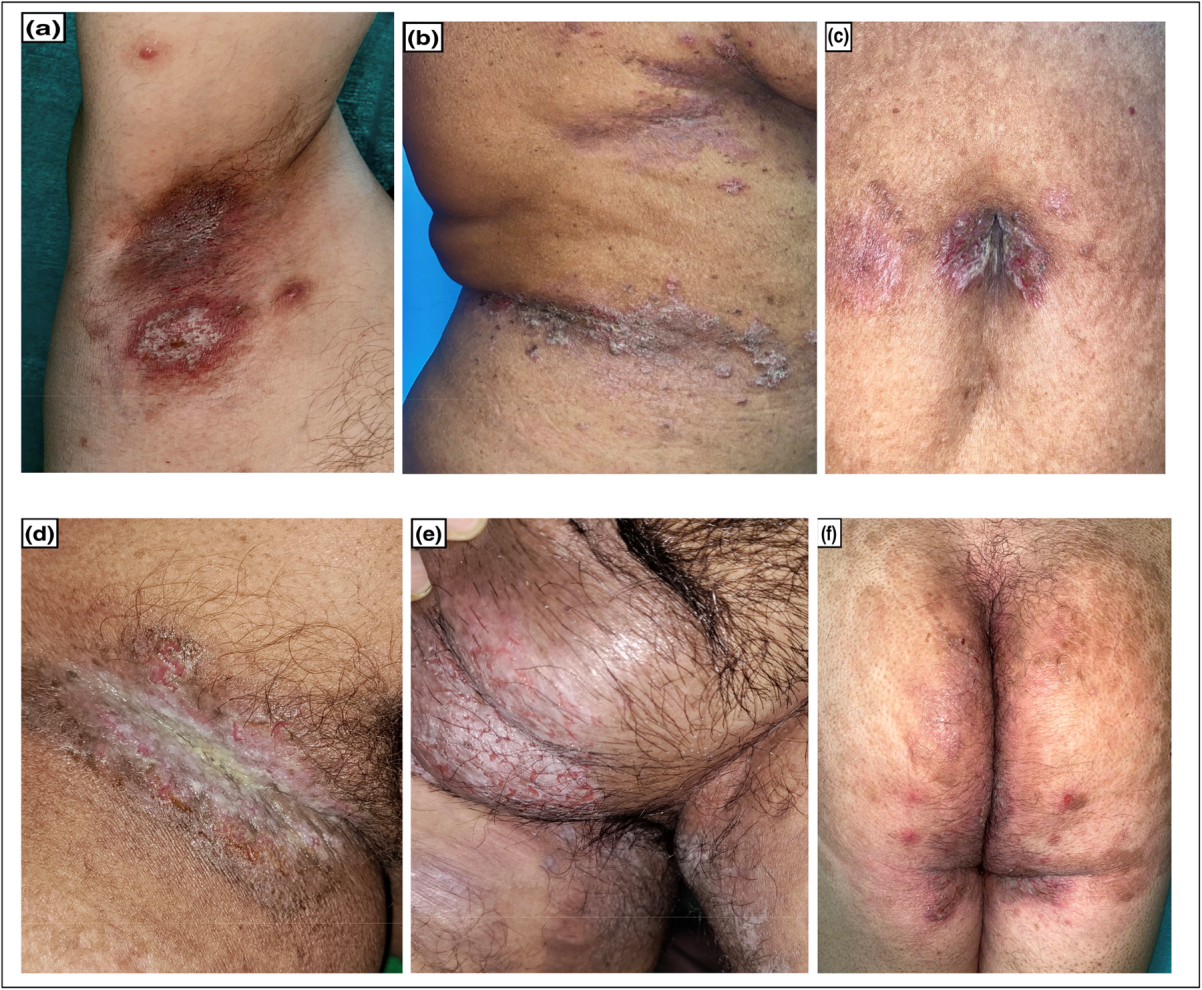
Ill‐defined erythematous plaque with fissuring and maceration over right axilla (a), sub mammary and waist region (b), umbilicus (c), right groin (d), scrotum (e) and perineal region and buttock (f).

The common sites of involvement in HHD are the neck, axillae, submammary folds, umbilicus, groin, and perineal region (Figure 1). The skin lesions are bilaterally symmetrical and may develop into erosions and crusts. Vegetative or verrucous plaques may be seen in chronic lesions. The scalp, antecubital, popliteal fossae, and vulvar regions are less commonly affected. The rare presentations of this dermatosis include segmental, extensive, mucosal, perianal, photoexposed area, or non‐intertriginous skin involvement [10]. The lesions are often associated with pain and itching, as well as an increased risk of squamous cell carcinoma [11]. Nail changes are seen in up to 70% of patients and are characterized by whitish longitudinal bands [8].

## 5 Histopathology

**Figure 2:**
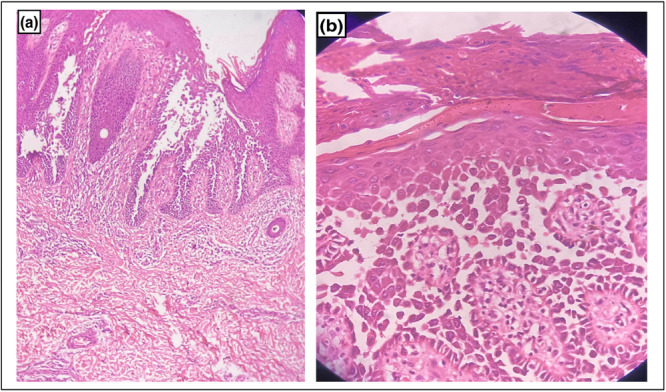
Haematoxylin and eosin staining revealed intraepidermal clefting and suprabasal acantholysis giving the appearance of “dilapidated brick wall” on 10X view (a) and 40X view (b). Overlying scale and crust along with parakeratosis can be seen (b).

Histopathologically, HHD is characterized by suprabasal acantholysis leading to bulla formation; however, the acantholysis is partial, the cell retaining some connections and giving an appearance of a ‘dilapidated brick wall’. Moderate perivascular lymphocytic infiltrates are seen in the superficial dermis (Figure 2). The adnexal structures and follicular epithelium are spared, and necrotic keratinocytes and acantholytic cells are rarely observed. Epidermal hyperplasia, parakeratosis, and crusting are seen in chronic lesions. Occasionally, dyskeratotic cells similar to the corps ronds and grains of Darier's disease are also seen. Direct immunofluorescence of the skin biopsy is negative [3, 8]. Immunohistochemical studies have shown that major desmosomal proteins and glycoproteins are synthesized in the uninvolved epidermis of HHD. In lesional skin, there is significant cytoplasmic labelling for the desmoplakins, desmogleins, and desmocollins [8].

## 6 Diagnosis

HHD can be diagnosed on clinical grounds; however, skin biopsy helps to confirm the diagnosis. Sequencing of the responsible gene, ATP2C1, is not routinely performed in clinical practice but may be useful in genetic counselling [8]. The differential diagnoses included intertrigo, tinea cruris, erythrasma, inverse psoriasis, pemphigus vegetans, Darier's disease, and Galli‐Galli disease [3].

## 7 Management

Evidence for treatments of HHD is limited to case reports, case series, and expert opinion [11]. We highlighted the various treatment modalities described for HHD in the literature with their proposed mechanism of action and summarized them in Tables 1, 2, 3.

**Table 1:** Topical therapeutic modalities with proposed mechanism of action in Hailey‐Hailey Disease.

Treatment modalities	Proposed mechanism of action in HHD	Level of evidence
Topical antimicrobials (clindamycin, gentamicin, mupirocin, Castellani paint and imidazoles) [8, 12, 13]	Antimicrobial propertyTopical gentamicin can promote translational read‐through of nonsense mutations in ATP2C1	Case report, retrospective study
Topical calcineurin inhibitors (Topical tacrolimus, pimecrolimus and cyclosporin) [3]	Anti‐inflammatory property	Case series
Topical steroids [3, 8]	Anti‐inflammatory property	Cross sectional study, case series
Topical 5‐flurouracil [14]	Restores normal intracytoplasmic calcium concentrations	Case reports
Iodine cadexomer [15]	Anti‐inflammatory, antimicrobial, and skin exudate absorptive properties	Case report
Topical vitamin D analogues (tacalcitol, calcitriol) [16]	Maintain the calcium gradient in differentiating keratinocytes, regulate and preserve the desmosome assembly and integrity	Case reports
Topical timolol and cinacalcet [17, 18]	Increase the intracellular calcium levels	Case reports
Topical ruxolitinib [19]	JAK kinase inhibitor; blockade of cytokine‐driven pathways and decrease inflammation	Case report
Topical combination of ketamine and diphenhydramine [20]	Antipruritic, antinociceptive, anaesthetic, and anti‐inflammatory effects	Case report
Topical aqueous aluminum chloride [21]	Block eccrine gland duct, produce atrophy and vacuolation of the secretory glandular cells	Case report

**Table 2:** Systemic therapeutic modalities with proposed mechanism of action in Hailey‐Hailey disease.

Treatment modalities	Proposed mechanism of action in HHD	Level of evidence
Oral antimicrobials (erythromycin, flucloxacillin, tetracyclines, dapsone) [11]	Antimicrobial, anti‐inflammatory property	Case series
Oral steroids [11]	Anti‐inflammatory property	Case series and retrospective study
Cyclosporine [22]	Anti‐inflammatory property	Case reports
Methotrexate [8, 23, 24]	Anti‐inflammatory and antiproliferative property	Case reports
Thalidomide [25]	Anti‐inflammatory and immunomodulatory property	Case reports
Oral retinoids (alitretinoin, acitretin, etretinate, isotretinoin) [26, 27]	Helps in cell proliferation, keratinization, differentiation, cellular adhesiveness, immunomodulation, and upregulation of ATP2CA1 gene expression	Case reports and case series
TNF‐ *α* inhibitors (adalimumab, etanercept) [28, 29]	Inhibit TNF‐ *α* and its effects on calcium homeostasis	Case report and case series
Dupilumab [30]	Inhibit IL‐4 and IL‐13 leading to promotion of the intracellular calcium signalling	Case report and case series
Tralokinumab [31]	IL‐13 inhibitor that increases influx of calcium into keratinocytes, helps in keratinocyte differentiations and cellular adhesion	Case report
Oral JAK kinase inhibitors (upadacitinib, atrocitinib) [32, 33]	Target Th2 cytokines like IL‐4, 13 which mediate their action via JAK/STAT pathway	Case reports
Apremilast [34]	Phosphodiesterase‐4 inhibitor, leading to inhibition of T_h_1 and T_h_17 response and decrease of CXCL10 release	Case reports
Low dose naltrexone [35]	Inhibit TLR‐4 that modulates the calcium transportation dysfunction and reduce IL‐6, TNF alpha and other inflammatory cytokines	Case report and case series
Afamelanotide [36]	Antioxidative effects	Case report
Magnesium chloride [37]	Act as an inhibitor of the Ca^2+^‐extruding activity in keratinocytes favouring intracellular Ca^2+^ accumulation and prevent acantholysis	Case reports
Liraglutide [38]	GLP 1 agonist, adjunctive therapy in HHD, helps in weight reduction	Case report
Glycopyrrolate [39]	Inhibits the sympathetic stimulation of eccrine sweat glands by blocking the M3 muscarinic receptors on glandular tissue, thereby reducing hyperhidrosis	Case reports

**Table 3:** Procedural and surgical therapeutic modalities with proposed mechanism of action in Hailey‐Hailey disease.

Treatment modalities	Proposed mechanism of action in HHD	Level of evidence
Botulinum toxin injection [40]	Cause chemodenervation by blocking the release of acetylcholine from the nerve terminals, which results in inhibition of sudoriferic nerves and reduction of sweat production by the eccrine glands.	Open level interventional study, systematic review, retrospective study, case series and case reports
Laser therapy [41, 42]	Ablates the epidermis while preserving most of the dermis and adnexal structures, leading to re‐epithelialization	Observational study, systematic review, retrospective study, case series and case reports
Photodynamic therpay [43, 44]	Intracellular protoporphyrin IX accumulation within the keratinocytes, which disrupts the cellular structures such as mitochondria, lysosomes, and endoplasmic reticulum	Case reports and case series
Narrowband Ultraviolet B therapy [45, 46]	Modulation of the intracellular Ca^2+^ homeostasis via cutaneous synthesis of the vitamin D	Case reports
Electron beam radiation therapy [47]	Direct ionization, which disrupts intracellular function, immunosuppression, and inhibit excessive epidermal cell proliferation	Case reports and case series
Dermabrasion [48, 49]	Leads to the destruction of the epidermis and superficial dermis while preserving the skin appendages, enabling re‐epithelialization of the treated area.	Case reports and case series
Surgical excision [50]	Removal of adnexal structures and the reduction in sweating and maceration	Case reports and case series

### 7.1 Nonpharmacological Treatment

HHD has a chronic relapsing and remitting course and is frequently aggravated by heat, sweating, trauma, sun exposure, infections, and pregnancy. General measures to reduce skin friction in the flexures, including weight loss, loose clothing, and absorbent pads in skin folds, are of paramount importance in limiting the relapses [8, 11].

### 7.2 Topical Treatments

The aim of the topical therapy is to control inflammation and to limit microbial colonization [8].

#### 7.2.1 Topical Antimicrobials

*Staphylococcus*, *Streptococcus*, *Candida* species, and dermatophytes are common colonizers in the skin lesions and may aggravate lesions or preclude healing. Various topical antimicrobials used include clindamycin (1%), gentamicin (0.1%), mupirocin (2%), Castellani paint, and clotrimazole applied two to four times a day for 2–4 weeks [8, 51]. In addition, gentamicin can induce readthrough of nonsense mutation in ATP2C1, the type of mutation seen in about 20% genotypes of HHD [8, 12, 13]. If lesions persist despite antibiotic and/or antifungal therapy, polymerase chain reaction‐based testing or viral culture for herpes simplex virus infection may be considered [8, 11].

#### 7.2.2 Topical Corticosteroids

Topical corticosteroids are used to modulate inflammation and are frequently combined with topical antimicrobial agents; however, their use is limited by side effects, particularly skin atrophy. As a general rule, they should be used for short duration at the lowest effective potency to treat an acute flare‐up of the disease. The skin lesions that do not respond to topical corticosteroids may benefit from a trial of intralesional steroids [3].

#### 7.2.3 Topical Calcineurin Inhibitors

Topical calcineurin inhibitors like tacrolimus (0.1%), pimecrolimus (1%), and cyclosporin are useful for long‐term control of inflammation, and unlike steroids, these agents do not lead to skin atrophy [3, 8]. Several case studies suggest the application of tacrolimus 0.1% ointment once or twice daily; though, topical use of tacrolimus itself can lead to superadded infections, including herpes simplex infection. A case in literature reports the development of squamous cell carcinoma following prolonged use of topical tacrolimus in vulvar HHD [52].

#### 7.2.4 Other Topical Agents

There are few case reports suggesting the beneficial effect of 5‐fluorouracil, iodine cadexomer, topical vitamin D analogues, topical timolol, topical cinacalcet, topical ruxolitinib, topical aluminum chloride, and topical combination of ketamine and diphenhydramine in HHD [14, 15, 16, 17, 18, 19, 20, 21]. It has been proposed that 5‐flurouracil (5%) cream restores normal intracytoplasmic calcium concentrations, explaining its efficacy in HHD [52]. Iodine cadexomer, a sterile formulation of 0.9% iodine bound to water‐soluble starch molecules, has antimicrobial, anti‐inflammatory, and skin exudate absorptive properties, which is beneficial in HHD [8, 14]. Topical vitamin D analogues (tacalcitol, calcitriol) are used in HHD, and the proposed mechanism is that they maintain the calcium gradient in differentiating keratinocytes and regulate and preserve the assembly and integrity of desmosomes in HHD [16].

Topical timolol (0.5%), a beta‐adrenergic blocker, and topical cinacalcet (3%), a calcium‐ sensing receptor agonist, are purported to increase the intracellular calcium levels [17, 18]. Similarly, topical ruxolitinib (1.5%), a JAK kinase inhibitor, when combined with biologics, has shown to be effective in HHD [19]. Topical combination of ketamine and diphenhydramine has been used to maintain remission in a case in which remission was induced by low‐dose naltrexone [20]. Topical 15% aqueous aluminium chloride solution is shown to be helpful in HHD in a recent report, as they reduce sweating by acting at the level of the eccrine gland duct, blocking it, and producing atrophy and vacuolation of the secretory glandular cells [21].

### 7.3 Systemic Therapy

Systemic treatments are often used when lesions do not respond to topical treatments or when the disease is too extensive to be treated topically [11].

#### 7.3.1 Oral Antimicrobials

The broad‐spectrum antibiotics like erythromycin, flucloxacillin, tetracyclines, and dapsone have been found to be effective in HHD. Painful, recalcitrant disease is suggestive of superinfection by herpes simplex, and systemic antiviral treatment should be considered [8, 11].

#### 7.3.2 Oral Steroids

Oral steroids have been used during flares or at low doses for maintenance therapy. However, these are not frequently used due to systemic side effects and rebound exacerbations upon discontinuation [11].

#### 7.3.3 Other Immunosuppressants

Several immunosuppressants like methotrexate, cyclosporine, thalidomide, and azathioprine have been used in recalcitrant disease with variable success rates [8, 11]. Cyclosporine helps to regulate intracellular calcium levels and pro‐inflammatory cytokine levels in keratinocytes. Low‐dose cyclosporine (2.5 mg/kg/day) for 3 weeks, with slow weaning over 6 months has maintained remission for 2 years in one patient [22]. Methotrexate, at a dose of 7.5–15 mg per week, has been shown to be effective in recalcitrant HHD [23, 24]. Thalidomide with immunomodulatory and anti‐inflammatory effects on TNF‐α and CD8 + T‐cells causes inhibition of Th1 inflammation, which was found beneficial in HHD; however, long‐term side effects should be discussed in depth before prescribing this medication [8, 25].

#### 7.3.4 Oral Retinoids

The recalcitrant lesions may be treated with oral retinoids, although their teratogenic effects must be considered when prescribing them to women of reproductive age. Oral retinoids may influence cell proliferation, differentiation, keratinization, cellular adhesiveness, immunomodulation, and upregulation of ATP2CA1 gene expression [26]. Several cases have shown positive results with retinoids like alitretinoin (30 mg/day), acitretin (10–25 mg/day), etretinate (25–60 mg/day) or isotretinoin (0.5 mg/kg/day); however, failure with retinoids has also been reported [8, 26, 27].

#### 7.3.5 Biologics

TNF‐α is thought to play a role in the pathogenesis of HHD through its effects on calcium homeostasis, so TNF‐*α* inhibitors like adalimumab and etanercept are seen as effective in HHD [28, 29]. Dupilumab, a monoclonal antibody directed against IL‐4 and IL‐13 possibly works in HHD as inhibition of IL‐4 and IL‐13 modulates favourably the intracellular calcium signaling [28, 30]. Tralokinumab, an IL‐13 inhibitor that increases the influx of calcium into keratinocytes, which is critical for normal keratinocyte differentiation and cellular adhesion, is found to be beneficial in HHD [31]. A case report showed control of skin lesions in a patient with multiple sclerosis and HHD who was treated with ocrelizumab (humanized anti‐CD20 monoclonal antibody) [53].

#### 7.3.6 Other Systemic Treatment

Lately, oral JAK kinase inhibitors like upadacitinib and atrocitinib have been reported to be useful in HHD as they target Th2 cytokines like IL‐4 and IL‐13, which mediate their action via the JAK/STAT pathway [32, 33]. Apremilast, a phosphodiesterase‐4 inhibitor, works by inhibiting Th1 and Th17 response and a decrease of CXCL10 release that is responsible for inflammation and flares in HHD [34]. Low‐dose naltrexone (1.5–6.25 mg/day) has been tried in HHD as it likely modulates the calcium transportation dysfunction by inhibiting TLR‐4, leading to further reduction in IL‐6, TNF‐α, and other inflammatory cytokines [20, 35]. Other systemic agents used in HHD with variable success are α‐melanocyte‐stimulating hormone (afamelanotide), magnesium chloride, vitamin D, liraglutide, glycopyrrolate, and oxybutynin [8, 36, 37, 38, 39].

### 7.4 Procedural Therapy

#### 7.4.1 Botulinum Toxin Injection

The protein botulinum toxin type A (BTA) causes chemodenervation by blocking the release of acetylcholine from the nerve terminals, which results in inhibition of sudoriferic nerves and reduction of sweat production by the eccrine glands. The decrease in sweating, and therefore humidity, provides protection against colonization by microorganisms and subsequent aggravation of HHD. BTA is an effective and safe treatment for HHD, particularly in intertriginous areas; however, it requires regular maintenance reinjections [40].

#### 7.4.2 Laser Therapy

Given the recurrent nature of HHD and the paucity of proven effective treatments, laser therapy has been studied, with CO_2_ laser in continuous mode showing the most promising results. The laser ablates the epidermis while preserving most of the dermis and adnexal structures, leading to re‐epithelialization and resolution of the lesions of the dermatoses. Traditional ablative lasers like the CO₂ and Er:YAG vaporize tissue by targeting water [41]. Er:YAG laser causes less scarring than CO₂ laser due to its higher water absorption and minimal thermal damage but is more superficial and lacks haemostatic effect [41, 42]. Fractional ablative laser therapy greatly minimizes the side‐effect profile observed with fully ablative laser therapy while obtaining positive results [42]. In contrast, non‐ablative lasers like diode laser and pulsed dye laser target specific chromophores such as melanin or haemoglobin, minimizing damage to surrounding tissue. However, their epidermal sparing might lead to decreased laser treatment efficacy compared to ablative lasers in HHD therapy [8, 42].

#### 7.4.3 Photodynamic Therapy

Photodynamic therapy can be considered in patients with disease refractory to multiple previous treatments; however, the results are conflicting [8, 43]. The mechanism by which PDT led to improvement in HHD is unknown but is believed to be due to intracellular accumulation of protoporphyrin IX within the epidermal keratinocytes, which disrupts the cellular structures such as lysosomes, mitochondria, and endoplasmic reticulum [44].

#### 7.4.4 Other Procedural Therapy

Narrowband Ultraviolet B therapy and electron beam radiation therapy have been tried in HHD, but further studies are required to evaluate their efficacy [45, 47]. The beneficial effect of NB‐UVB phototherapy may also be mediated through the modulation of the intracellular Ca^2+^ homeostasis via cutaneous synthesis of the vitamin D [46]. The success of electron beam radiation therapy in HHD is likely secondary to direct ionization, which disrupts intracellular function, immunosuppression, and inhibits excessive epidermal cell proliferation [47].

### 7.5 Surgical Therapies

#### 7.5.1 Dermabrasion

Dermabrasion leads to the destruction of the epidermis and superficial dermis while preserving the skin appendages, enabling re‐epithelialization of the treated area. Dermabrasion is a treatment option for recalcitrant HHD that does not respond to conservative therapy [48, 49].

#### 7.5.2 Surgical Excision

Surgical excision of the affected areas followed by split‐thickness skin grafting is occasionally performed, especially for refractory cases that is unresponsive to conservative treatments. The removal of adnexal structures and the reduction in sweating and maceration are likely to contribute to the success of surgical excision in HHD [50].

## 8 Conclusion

HHD is a genodermatosis that has no cure. While the basic defect in HHD is known and the resultant pathology is that of a decrease in intracellular calcium levels, drugs that address this pivotal defect are being investigated, and the best therapeutic option for patients with HHD is yet to be identified. Thus, currently the main aim of treatment is to control symptoms and reduce recurrence so that the patient's quality of life can be improved.

## Author Contributions

**Mahesh Mathur:** conceptualization, visualization, formal analysis, resources, supervision, validation, writing – original draft. **Sumit Paudel:** conceptualization, formal analysis, resources, supervision, validation, visualization. **Nabita Bhattarai:** data curation, investigation, visualization, writing – review and editing. **Sambidha Karki:** investigation, visualization, data curation, writing – review and editing. **Sandhya Regmi:** conceptualization, writing – original draft, validation, formal analysis, resources, supervision, visualization. All authors have read and approved the final version of the manuscript. I, Dr. Sandhya Regmi, had full access to all of the data in this study and takes complete responsibility for the integrity of the data and the accuracy of the data analysis.

## Ethics Statement

Reviewed and approved by Institutional review board College of medical sciences (IRBCOMS).

## Consent

The patients in this manuscript have given written informed consent to the publication of their case details.

## Conflicts of Interest

The authors declare no conflicts of interest.

## 1 Transparency statement

I, Dr. Sandhya Regmi, affirms that this manuscript is an honest, accurate, and transparent account of the study being reported; that no important aspects of the study have been omitted; and that any discrepancies from the study as planned (and, if relevant, registered) have been explained.

## Data Availability

The data that support the findings of this study are available from the corresponding author upon reasonable request.
